# Supplementary material for: Ice sheet and precession controlled subarctic Pacific productivity and upwelling over the last 550,000 years
Source: Nat Commun. 2024 Apr 25;15:3489. doi: 10.1038/s41467-024-47871-8 (PMC11045732; doi:10.1038/s41467-024-47871-8)
Supplement: Supplementary file 1 — Supplementary information [file 41467_2024_47871_MOESM1_ESM.pdf]

**Supplementary material for**

# Ice sheet and precession controlled subarctic Pacific productivity and upwelling over the last 550,000 years


Zhengquan Yao[1,2]*†, Xuefa Shi[1,2]*†, Qiuzhen Yin[3]*, Samuel Jaccard[4], Yanguang Liu[1,2], Zhengtang Guo[5], Sergey A. Gorbarenko[6], Kunshan Wang[1,2], Tianyu Chen[7], Zhipeng Wu[3], Qingyun Nan[8], Jianjun Zou[1,2], Hongmin Wang[1,2], Jingjing Cui[1,2], Anqi Wang[1,7], Gongxu Yang[1], Aimei Zhu[1,2], Alexander Bosin[6], Yuri Vasilenko[6], Yonggui Yu[1,2]

[1] Key Laboratory of Marine Geology and Metallogeny, Shandong Key Laboratory of Deep-Sea Mineral Resources Development, First Institute of Oceanography, MNR, Qingdao, China

[2] Laboratory for Marine Geology, Qingdao Marine Science and Technology Center, Qingdao, China

[3] Earth and Climate Research Center, Earth and Life Institute, Université catholique de Louvain, Louvain-la-Neuve, Belgium

[4] Institute of Geological Sciences, University of Lausanne, Lausanne, Switzerland

[5] Key Laboratory of Cenozoic Geology and Environment, Institute of Geology and Geophysics, Chinese Academy of Sciences, Beijing, China

[6] V.I. Il'ichev Pacific Oceanological Institute, Far East Branch of Russian Academy of Science, Vladivostok, Russia

[7] State Key Laboratory for Mineral Deposits Research, School of Earth Sciences and Engineering, Nanjing University, Nanjing, China

[8] Key Laboratory of Marine Geology and Environment, Institute of Oceanology, Chinese Academy of Sciences, Qingdao, China

†These authors contributed equally to this work.

*Corresponding author: Zhengquan Yao (yaozq@fio.org.cn), Xuefa Shi (xfshi@fio.org.cn), Qiuzhen Yin (qiuzhen.yin@uclouvain.be)


**This PDF file includes:**

Supplementary Figures 1 to 7

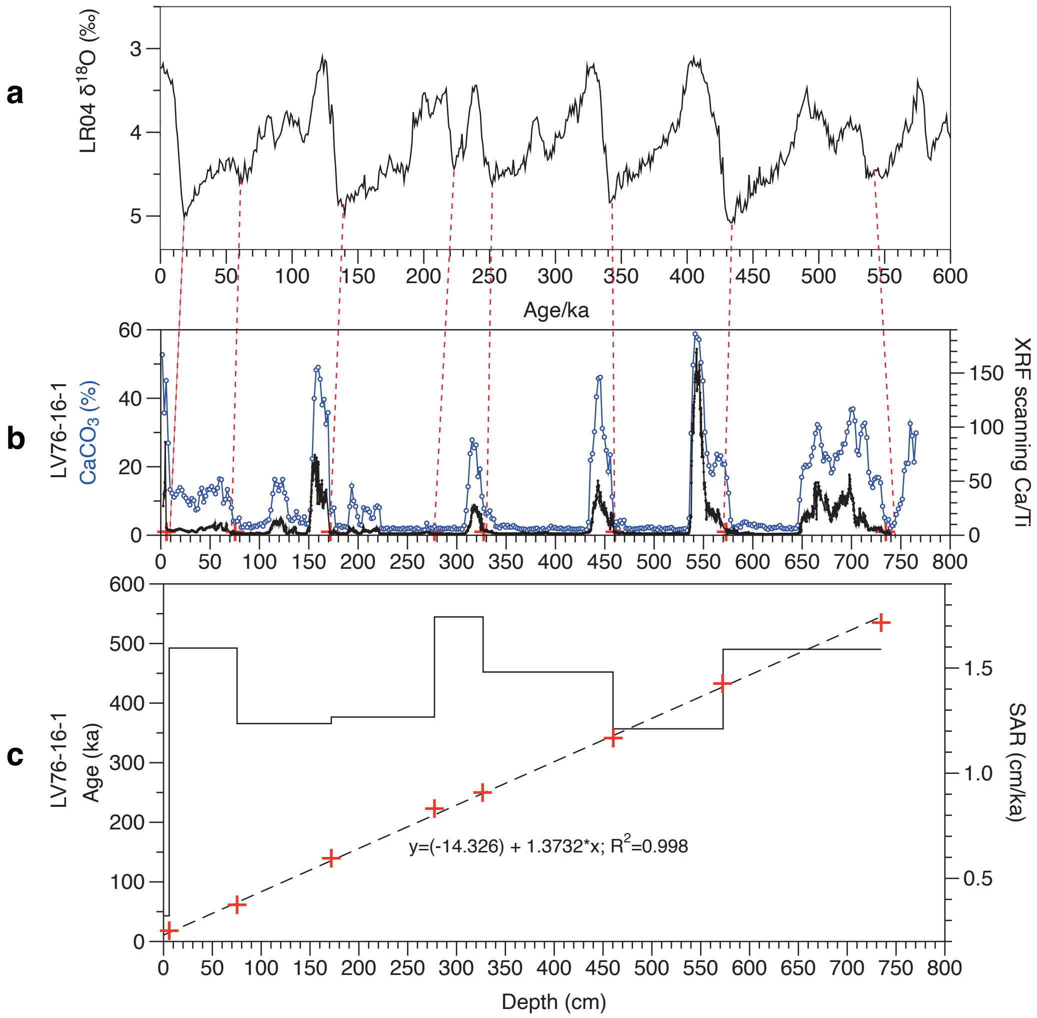


**Supplementary Fig. 1. The Age model for core LV76-16-1. (a)** Marine oxygen isotope stack (LR04)[20]. **(b)** Comparison of Ca/Ti and $CaCO_3$ content (circles) of core LV76-16-1. **(c)** Depth-age relation and sedimentary accumulation rate (SAR) for core LV76-16-1. A total of eight age points (red crosses) were obtained by correlating the abrupt increases in Ca/Ti to each major glacial termination, shown by red dashed lines. The black dashed line displays the linear correlation between age and depth. Source data are provided as a Source Data file.

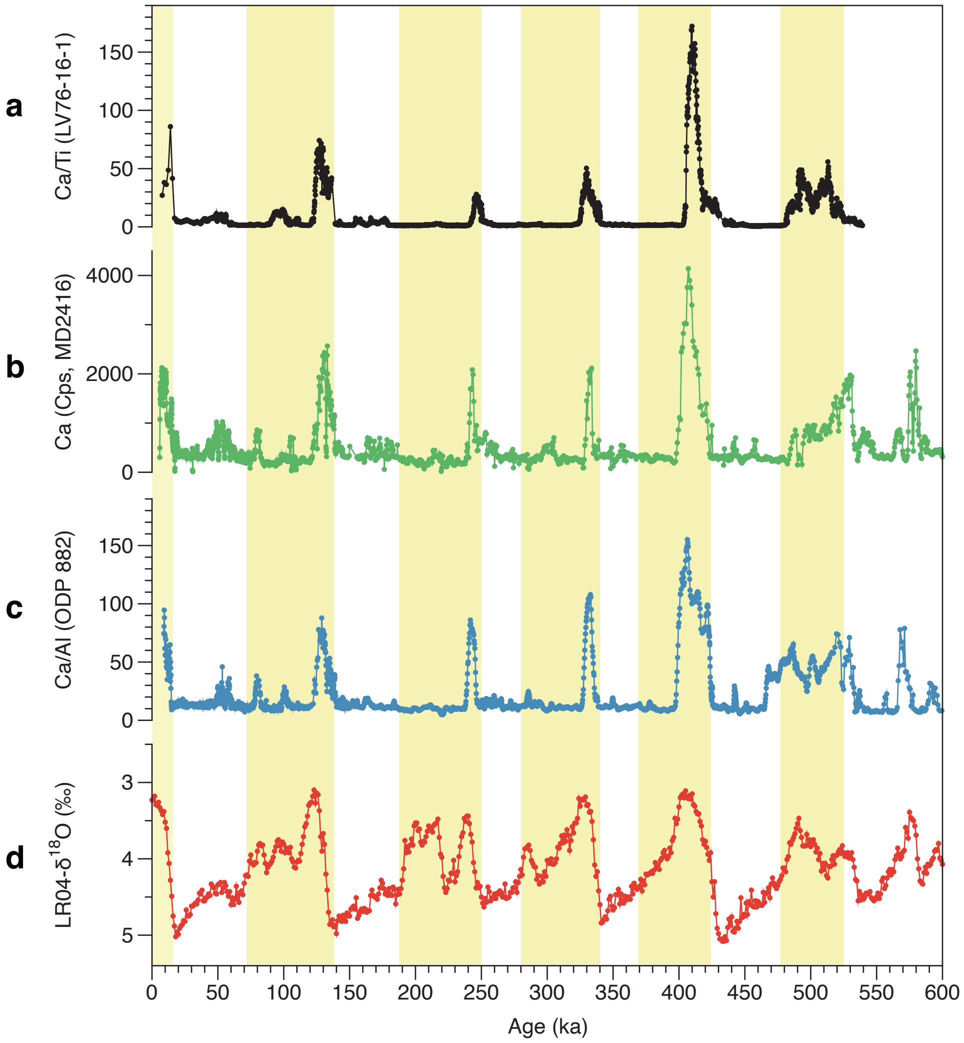


**Supplementary Fig. 2. Comparison of Ca/Ti in core LV76-16-1 with other records.** (a) Ca/Ti ratio of core LV76-16-1 (this study). (b) X-ray fluorscence (XRF) scanning Ca counts in core MD2416 in the NW Pacific[22]. (c) Ca/Al ratio at ODP Site 882 in the NW Pacific[12]. (d) Marine benthic $\delta^{18}O$ stack (LR04)[20]. The vertical yellow bars represent interglacial periods.

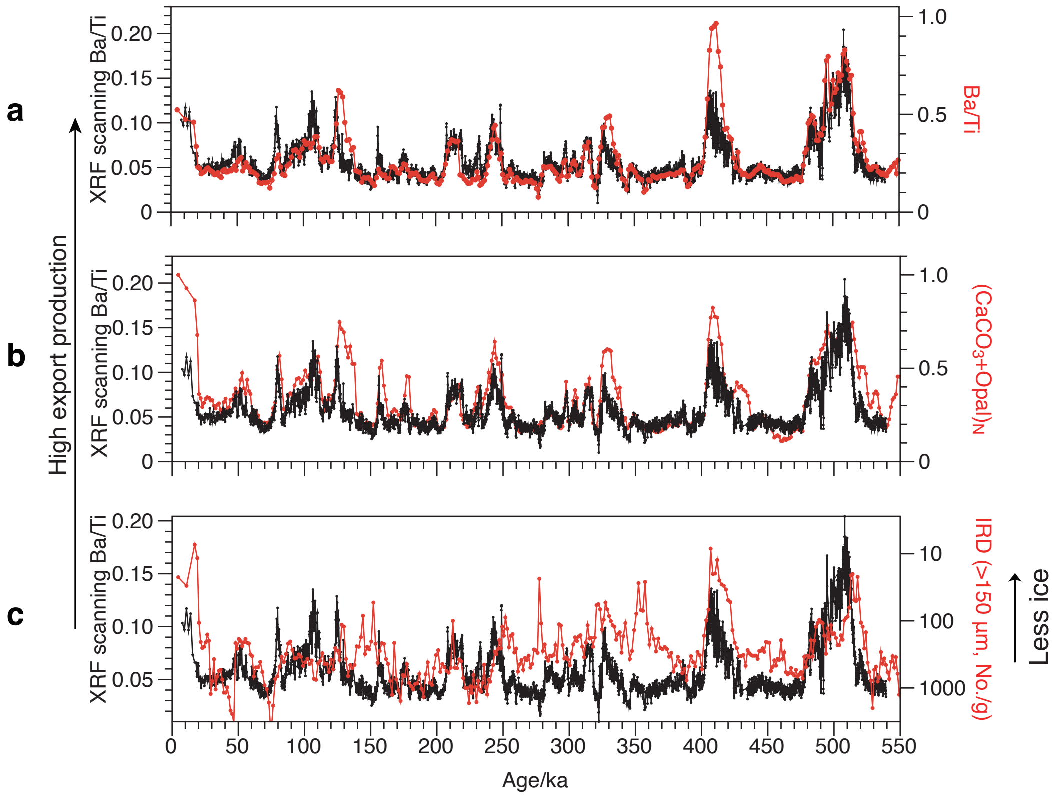


**Supplementary Fig. 3. Comparison of export production with other environmental proxies in core LV76-16-1. (a)** Comparison of X-ray fluorescence (XRF) scanning Ba/Ti with Ba/Ti ratio measured on discrete samples (red dots). **(b)** Comparison of XRF scanning Ba/Ti with the normalized sum of the $CaCO_3$ and opal contents (red dots). **(c)** Comparison of XRF scanning Ba/Ti with ice-rafted debris (IRD; red dots) indicated by the abundance of coarse grains (> 150 μm) in samples. Please note that the IRD abundance is plotted upside down. Source data are provided as a Source

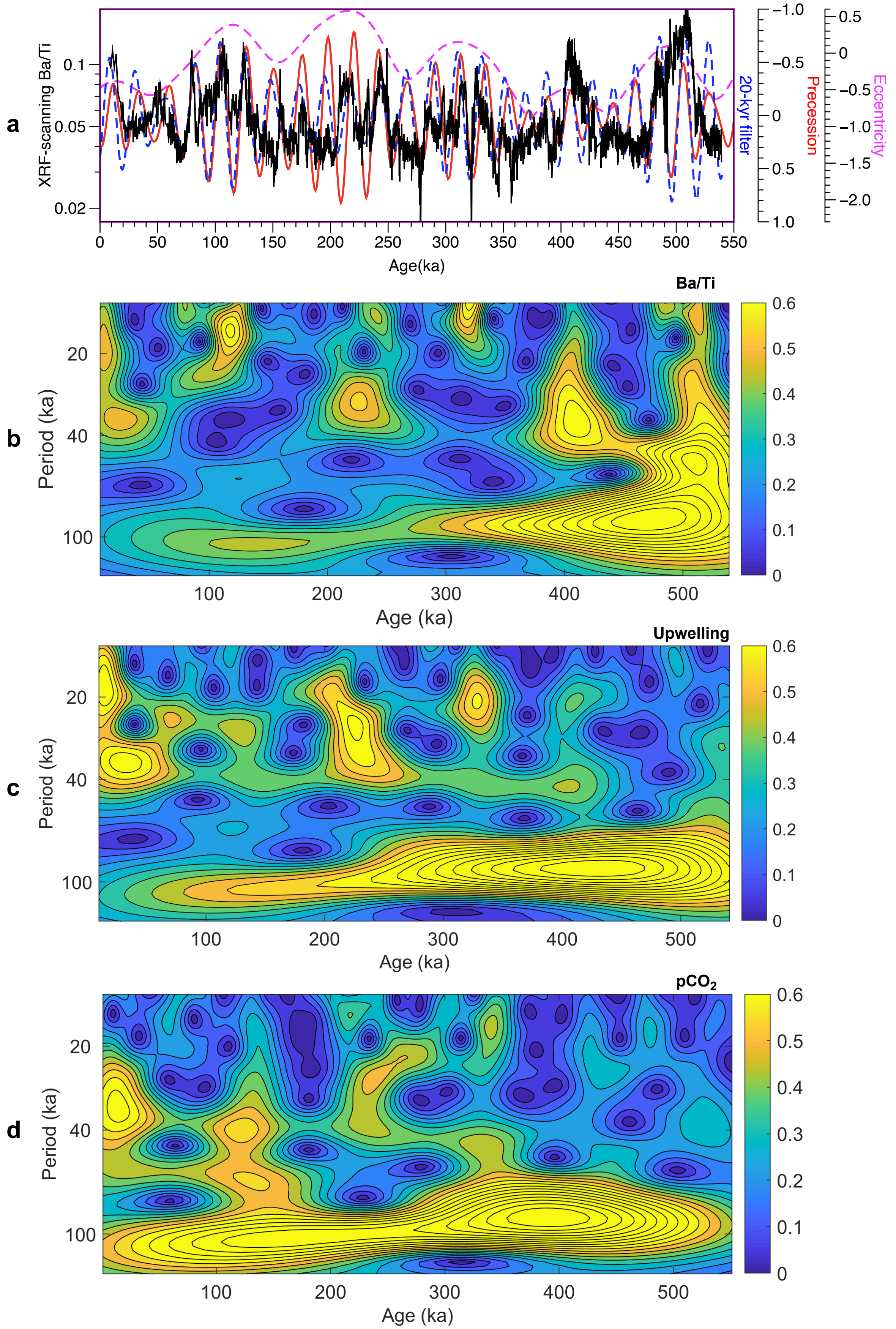


**Supplementary Fig. 4. Variations and wavelet analysis of Ba/Ti, upwelling index and atmospheric $CO_2$ concentrations ($pCO_2$). (a)** Comparison between Ba/Ti (dark line), 20-kyr filtering (blue line), Precession (red line) and Eccentricity (purple line)[60]. **(b)** Wavelet analysis of Ba/Ti from core LV76-16-1. **(c)** Wavelet analysis of upwelling index from core LV76-16-1. **(d)** Wavelet analysis of $pCO_2$[1,66].

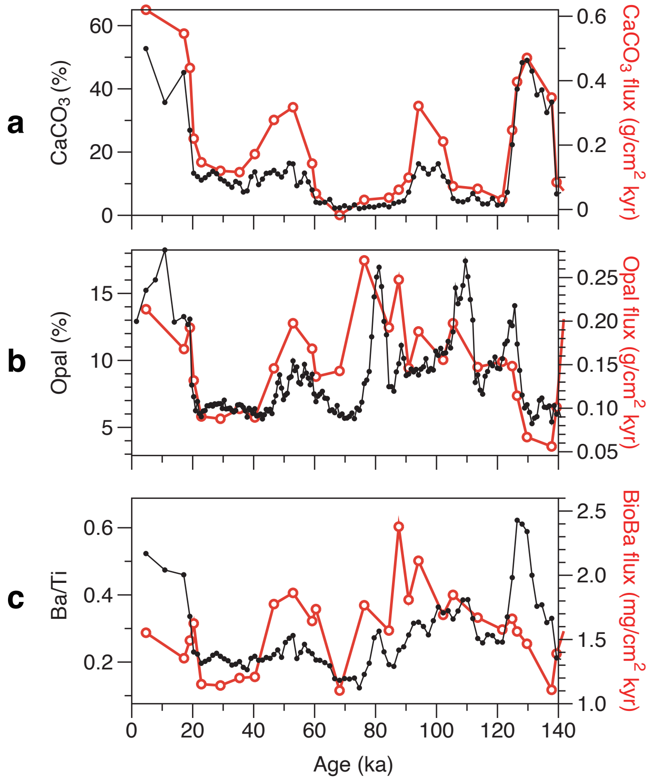


**Supplementary Fig. 5. $^{230}$Th-normalized biogenic particle flux for core LV76-16-1 over the last glacial cycle. (a)** Comparison between $CaCO_3$ content and $CaCO_3$ flux (red circles). **(b)** Comparison between opal content and opal flux (red circles). **(c)** Comparison between Ba/Ti and BioBa flux (red circles).

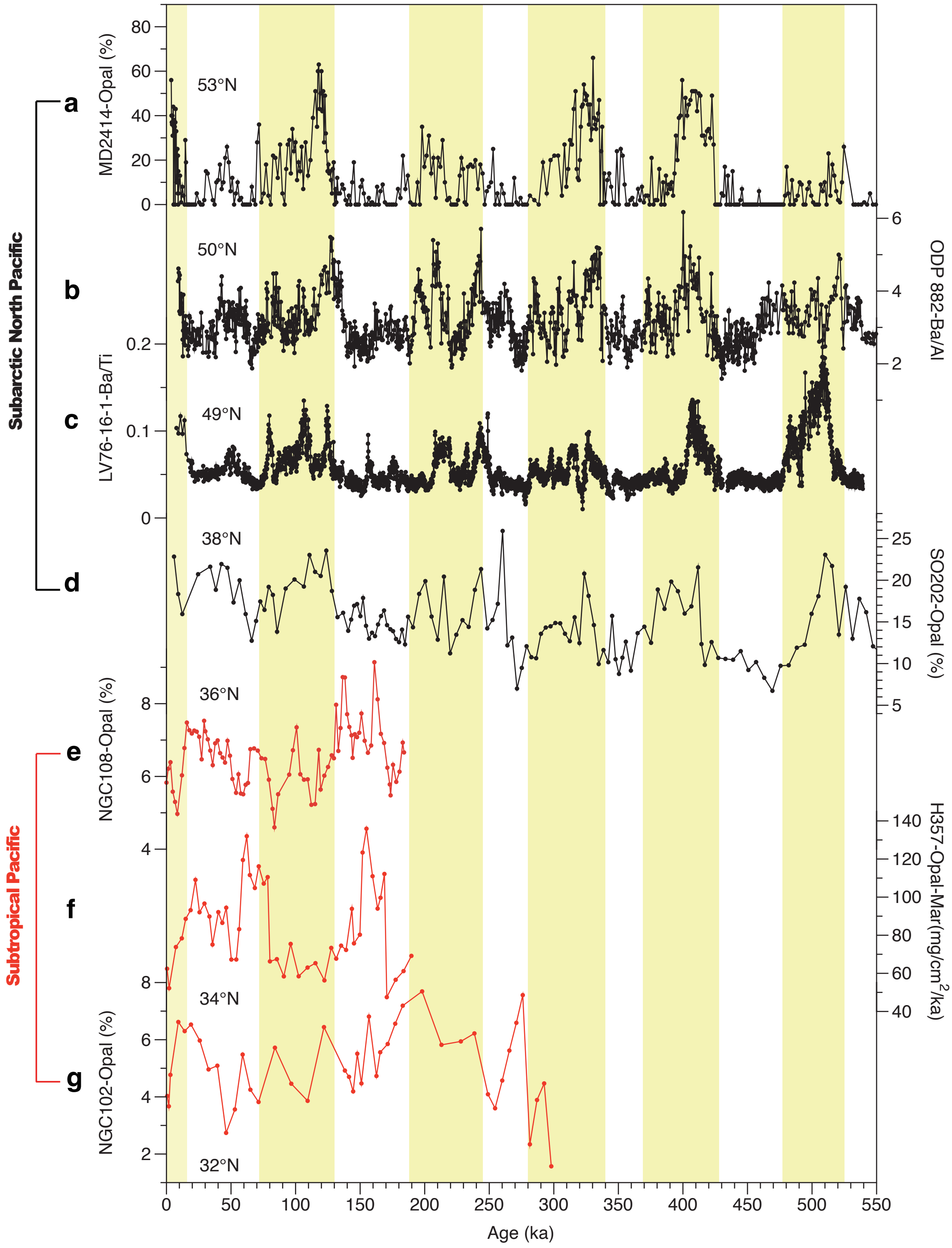


**Supplementary Fig. 6. Comparison of productivity records from different regions.** **(a-d)** Productivity from LV76-16-1 and other records from the Subarctic Pacific[11,29,30]. **(e-g)** Productivity records from the subtropical Pacific Ocean[40-42]. The vertical yellow bars represent interglacial periods.

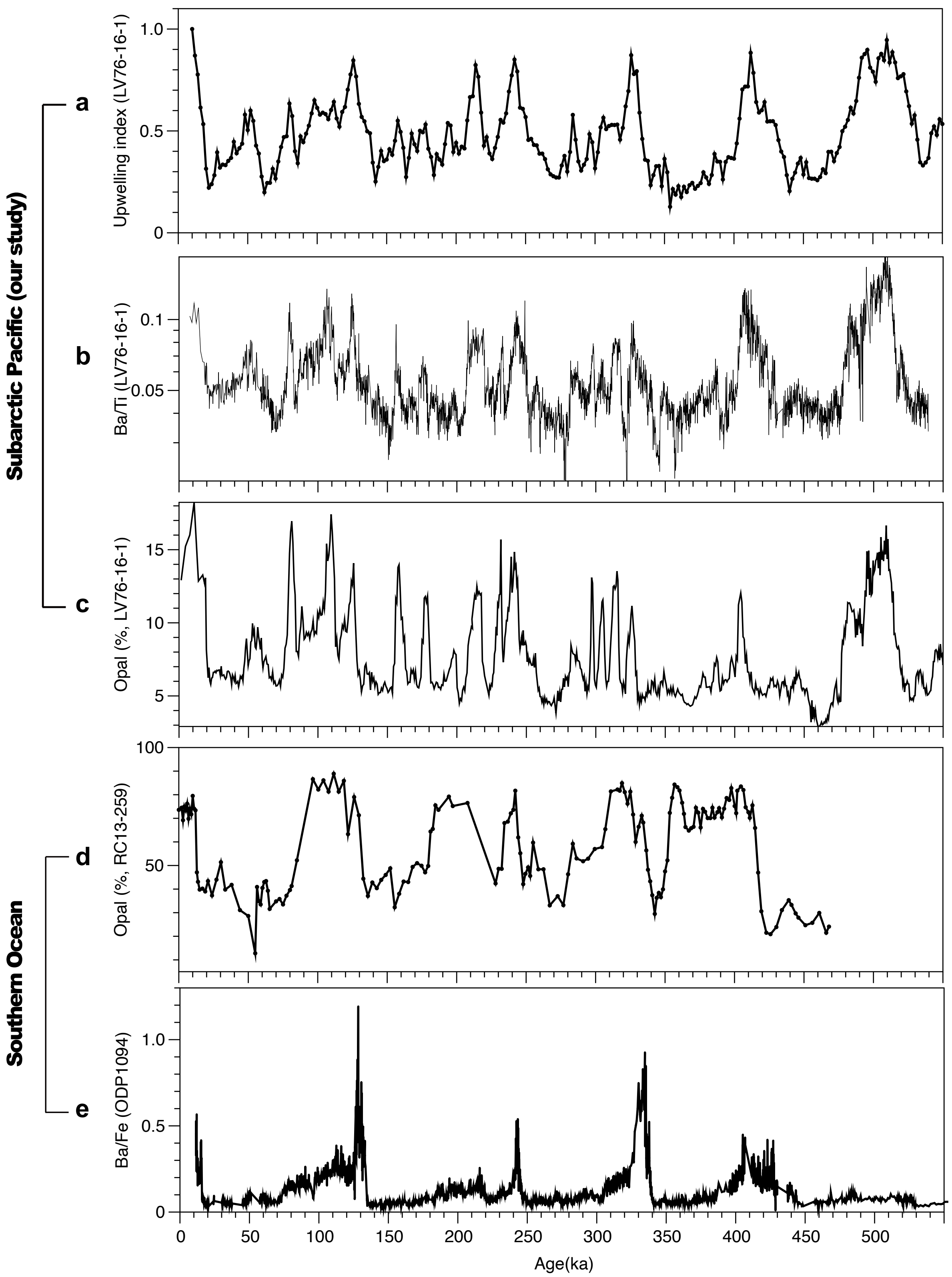


**Supplementary Fig. 7. Comparison between productivity, upwelling proxies of Core LV76-16-1 and records from the Southern Ocean.** (a) Variations in upwelling index of LV76-16-1. (b) Variations in Ba/Ti of LV76-16-1. (c) Variations in opal of LV76-16-1. (d) Variations in opal of Core RC13-259 from the Southern Ocean[52]. (e) Variations in Ba/Fe of ODP Site 1094 from the Southern Ocean[4].
